# Microbial community-based protein from soybean-processing wastewater as a sustainable alternative fish feed ingredient

**DOI:** 10.1038/s41598-024-51737-w

**Published:** 2024-01-31

**Authors:** Ezequiel Santillan, Fanny Yasumaru, Ramanujam Srinivasan Vethathirri, Sara Swa Thi, Hui Yi Hoon, Diana Chan Pek Sian, Stefan Wuertz

**Affiliations:** 1 Singapore Centre for Environmental Life Sciences Engineering, Nanyang Technological University, Singapore, 637551 Singapore; 2 Aquaculture Innovation Centre, Temasek Polytechnic, Singapore, 529757 Singapore; 3 School of Civil and Environmental Engineering, Nanyang Technological University, Singapore, 639798 Singapore

**Keywords:** Environmental biotechnology, Environmental sciences, Environmental microbiology, Next-generation sequencing, Animal physiology

## Abstract

As the global demand for food increases, aquaculture plays a key role as the fastest growing animal protein sector. However, existing aquafeeds contain protein ingredients that are not sustainable under current production systems. We evaluated the use of microbial community-based single cell protein (SCP), produced from soybean processing wastewater, as a partial fishmeal protein substitute in juvenile Asian seabass (*Lates calcarifer*). A 24-day feeding trial was conducted with a control fishmeal diet and a 50% fishmeal replacement with microbial community-based SCP as an experimental group, in triplicate tanks containing 20 fish each. Both diets met the protein, essential amino acids (except for lysine), and fat requirements for juvenile Asian sea bass. The microbial composition of the SCP was dominated by the genera *Acidipropionibacterium* and *Propioniciclava*, which have potential as probiotics and producers of valuable metabolites. The growth performance in terms of percent weight gain, feed conversion ratio (FCR), specific growth rate (SGR), and survival were not significantly different between groups after 24 days. The experimental group had less variability in terms of weight gain and FCR than the control group. Overall, microbial community-based protein produced from soybean processing wastewater has potential as a value-added feed ingredient for sustainable aquaculture feeds.

## Introduction

It is estimated that 1250 million tons of animal-derived protein would need to be produced per year to meet the global demand in 2050 at current consumption levels^1^. However, increasing meat production for this purpose is not sustainable because the conversion of plant to meat protein is inefficient^2^. Protein production by conventional agriculture-based food supply chains is also a major issue in terms of global environmental pollution. The livestock sector produces 14.5% of the global anthropogenic greenhouse gas (GHG) emissions, and about 33% of the land surface and 75% of freshwater resources are used for livestock or crop production^3^. Thus, new solutions for protein production are needed to ensure sustainable food security.

The supply of seafood, wild-caught and farmed, for human consumption was around 175 million tons in 2020, making it the largest protein industry worldwide^4,5^. Aquaculture accounted for about 50% of that sector and 21% of total animal protein production^6^. With the global human population predicted to increase by 30% in 2050 compared to 2018, it is estimated that aquaculture production would need to increase by 57.2% to meet the growing demand for seafood^5^. High trophic level aquaculture species still rely on fishmeal and fish oil derived from wild capture fisheries^7–9^, highlighting the need for alternatives to fishmeal as a protein source in aquaculture.

Protein can be produced through the cultivation of various microbes, which is generally denoted as microbial protein or single cell protein (SCP)^10^. A recent life cycle assessment (LCA) showed a lower environmental impact of SCP production compared to that of soybean meal manufacturing^11^. SCP represents a promising alternative to fishmeal as a protein source in aquaculture^4^, as it does not compete with the human food supply. In fact, it can transform the so-called “organic waste” from the human food industry into a valuable and sustainable animal feed ingredient. Thus, it may offer solutions for a wide variety of products and production approaches in aquaculture, but considerable research, development, and scale-up efforts are required to reach its potential^12,13^.

In particular, SCP grown on food processing wastewaters (FPWW) holds promise as a sustainable source of protein in animal feed^14^. Collectively, FPWW possess attractive features for SCP production, such as a continuous global production of process water rich in dissolved carbon (C), nitrogen (N) and phosphorus (P) compounds^15^. These wastewaters are also considered to be free of pathogens, heavy metals, and other harmful contaminants. FPWW can support microbial growth in bioreactors, from which cells can be recovered and dried leading to products suitable for animal feed ingredients, while concurringly avoiding the cost of treating such wastewaters, and hence represent an important step toward a circular bioeconomy^16^.

Traditionally, SCP production involves the use of single strains in axenic cultures, incurring high initial and operational costs. For example, a recent study used pure and enriched cultures of purple bacteria as a protein ingredient (5–11% protein replacement) in whiteleg shrimp nursery feed^17^. A promising alternative would be a whole-community approach for SCP production, leveraging the microorganisms already present in the wastewater^14^. Comparatively, microbial community-based SCP reduces the need for costly specific growth media, as well as the overall energy input required for SCP production. Microbial communities can be more robust and resilient to process fluctuations, leading to more stable SCP production. Further, the use of a microbial community may result in a more diverse and nutritionally rich SCP, as various microorganisms contribute different amino acids, vitamins, and other essential nutrients to the final product. A recent study showed that microbial community-based SCP produced directly from soybean processing wastewaters contained essential amino acids for fish as well as taxa with probiotic potential^18^. However, the feasibility of using such SCP as fish feed ingredient for aquaculture has yet to be established. Hence, the aim of this study was to evaluate the use of microbial community-based SCP, produced from soybean processing wastewater, as a value-added alternative ingredient to fishmeal for juvenile Asian seabass (*Lates calcarifer*) aquaculture.

## Materials and methods

### Fish source and experimental culture conditions

Unvaccinated Asian sea bass (*Lates calcarifer*, also known as barramundi) juveniles were obtained from a commercial hatchery (Allegro Aqua, Singapore). Fish were fed with a commercial sea bass diet (43% protein, Uni-President, Vietnam) for a period of 3 weeks to acclimate the fish to lab conditions prior to the start of the 24-day feeding trial. Juvenile sea bass of 19.7 ± 2.6 g were randomly distributed in rectangular glass aquaria (0.58 m × 0.58 m × 0.38 m, 100-L water volume) at a stocking density of 20 fish per aquaria. Subsequently, three aquaria were randomly assigned to each dietary treatment (n = 3). The glass aquaria were connected to a recirculating system with mechanical and biological filtration and aeration was supplied to each aquarium. Water temperature ranged from 27 to 29 °C. Other water quality parameters, i.e., dissolved oxygen (> 4 mg L^−1^), pH (7.0–7.9), salinity (15 ppt), ammonia (< 0.5 ppm) and nitrite (< 0.5 ppm) were kept within acceptable levels for the species. The study was approved by the Institutional Animal Care and Use Committee (IACUC) under number 202003-155A. All experiments were performed in accordance with relevant guidelines and regulations.

### Experimental diets and feeding protocol

A control fishmeal-based (FM) diet and an experimental 50% fishmeal replacement diet with microbial community-based SCP (50SCP), were formulated to contain at least 45% protein and 12% fat (Table 1). These were prepared with 30% fishmeal (FM) and with 50% replacement of the fishmeal with SCP (50SCP), while adjusting the other ingredients to maintain the crude protein and fat levels (Table 1). The nutritional composition of both diets is detailed in Table 2. Diet preparation involved mixing the dry feed ingredients first, followed by the addition of the liquids, including 20% v/v water, in a stand mixer (Model 5 QT, KitchenAid, St Joseph, MI, USA). The resulting mash was passed through a meat grinder attached to the stand mixer and fitted with a 3-mm diameter die. The moist strings were dried overnight in a ventilated drying oven at 45 °C. The dry strings were hand-broken and stored in air-tight plastic containers at 4 °C until used. Both diets were fed twice a day (0900 h, 1600 h) to apparent satiation, and feed intake was measured daily. Fish were group-weighed at the beginning and individually at the end of the 24-day feeding trial.

**Table 1:** Formulation of the fishmeal (FM) control diet and microbial community-based single-cell protein experimental diet (50SCP – 50% fishmeal replacement) used in the feeding trial with Asian sea bass (*Lates calcarifer*).

Ingredient (% w/w)	FM	50SCP
Fishmeal	30.0	15.0
Microbial community-based single-cell protein (SCP)	0.0	15.0
Poultry by-product meal	13.0	16.0
Squid liver meal	10.0	10.0
Dried yeast	3.0	3.0
Fish hydrolysate	5.0	5.0
Blood meal	4.0	4.0
Wheat flour	18.5	15.5
Wheat gluten	5.0	5.0
Soy lecithin	2.5	2.5
Fish oil	8.0	8.0
Vitamin premix	0.2	0.2
Mineral premix	0.2	0.2
Taurine	0.5	0.5
Ascorbic 2-phosphate 35%	0.2	0.2

**Table 2:** Nutritional composition (“as is” basis) of the fishmeal (FM) control diet and the experimental diet with 50% fishmeal replacement by microbial community-based single-cell protein (50SCP) used in the feeding trial with Asian sea bass (*Lates calcarifer*).

Composition	FM	50SCP	Requirement^†^	Method
Proximate composition (%)				
Moisture	6.5	6.4	–^#^	L
Crude protein (N × 6.25)	50.0	47.8	–	E
Crude fat	17.9	17.1	–	I
Crude Fibre	< 0.1	0.17	–	D
Ash	13.5	9.4	–	L
Amino acid composition (mg/100 g)				
Alanine	2590	2690	NA*	F
Arginine	2580	2550	1940	F
Aspartic acid	4760	4740	NA	F
Glutamic acid	6830	6600	NA	F
Glycine	2790	2730	NA	F
Histidine	1040	1040	820	F
Isoleucine	1650	1700	1260	F
Leucine	3280	3350	2300	F
Lysine	2180	2290	2660	F
Methionine	1020	943	870	F
Phenylalanine	1880	2020	1310	F
Proline	2850	2680	NA	F
Serine	2240	2220	NA	F
Threonine	1790	1830	1450	F
Tyrosine	1320	1300	1040	F
Valine	2090	2170	1500	F
Tryptophan	444	485	270	G
Total amino acids	41,656	41,488	NA	F
Vitamin composition				
Vitamin A (mg kg^−1^)	1.43	1.50	–	A
Vitamin D3 (IU kg^−1^)	1130	296	–	C
Vitamin B12 (µg kg^−1^)	11.6	139.8	–	B
Mineral composition				
Selenium (µg/100 g)	240	175	–	J
Calcium (mg/100 g)	2480	1800	–	K
Iron (mg/100 g)	37.7	49.7	–	K
Phosphorus (mg/100 g)	1750	1340	–	K
Zinc (mg/100 g)	18.1	24.6	–	K
Fatty acid composition (mg/100 g)				
Alpha linolenic C18:3n-3	243	239	–	H
Arachidic C20:0	78.2	75.7	–	H
Arachidonic C20:4n-6	141	109	–	H
Docosanoic C22:0	36.2	34.6	–	H
Butyric C4:0	6.32	6.95	–	H
Capric C10:0	6.73	18.3	–	H
Caproic C6:0	4.18	4.48	–	H
Caprylic C8:0	4.66	20.4	–	H
Dihomo-gamma-linolenic C20:3n-6	12.1	10.4	–	H
Docosahexaenoic C22:6n-3 (DHA)	657	463	–	H
Eicosadienoic C20:2n-6	9.84	8.34	–	H
Eicosapentanaeoic C20:5n-3 (EPA)	322	252	–	H
Eicosatrienoic C20:3n-3	8.74	6.87	–	H
Eicosenoic C20:1 (total)	69.7	60.9	–	H
Eicosenoic(Gondoic) C20:1n-9	69.7	60.9	–	H
Erucic acid C22:1n-9	9.82	9.08	–	H
Gamma linolenic C18:3n-6 (GLA)	5.19	5.7	–	H
Heptadecenoic C17:1n-7	22.9	22	–	H
Lauric C12:0	21.6	20.2	–	H
Lignoceric C24:0	33.8	30.8	–	H
Total linoleic acid	2800	2840	–	H
Linoleic C18:2n-6	2800	2840	–	H
Margaric C17:0	89.1	84.5	–	H
Monounsaturated fat (g/100 g)	5.7	5.64	–	H
Myristic C14:0	370	307	–	H
Myristoleic C14:1n-5	10.3	6.19	–	H
Nervonic C24:1n-9	25.8	19.8	–	H
Oleic C18:1n-9	5190	5210	–	H
Palmitic C16:0	5390	5070	–	H
Palmitoleic C16:1n-7	371	306	–	H
Pentadecanoic C15:0	54.4	57.4	–	H
Pentadecenoic C15:1n-5	6.33	9.67	–	H
Polyunsaturated fat (g/100 g)	4.23	3.96	–	H
Saturated fat (g/100 g)	7.13	6.69	–	H
Stearic C18:0	1020	948	–	H
Total fatty acids (g/100 g)	17.1	16.3	–	H
Total Omega 3 (g/100 g)	1.23	0.96	–	H
Total Omega 6 (g/100 g)	2.96	2.96	–	H
Total Omega 9 (g/100 g)	5.3	5.3	–	H

^†^Estimated dietary amino acid requirement profile of fish at a 45% dietary protein level, as percentage of dry diet, according to Tacon^42^.

*Not an essential amino acid.

^#^Not known.

^A^Methods for the Determination of Vitamins in Food—Recommended by COST 91 (1986).

^B^In-House No. F148 (Based on ELISA method).

^C^AsureQuality Method 5547, HPLC.

^D^In-house No. F1 (Based on Pearson's Chemical Analysis of Foods, 7th Ed., Pg. 16–18).

^E^AsureQuality Method 6503s, Kjeldahl Block Digestion.

^F^Analytical Biochemistry 178 (1989) (Modified).

^G^AsureQuality Method 5707, HPLC.

^H^AOAC 991.39.

^I^AsureQuality Method 4988, Gravimetric-SBR.

^J^AsureQuality Method 6311s, ICP-MS.

^K^AsureQuality Method 6324s, ICP-OES.

^L^AsureQuality Method 4208, Oven Dried.

### Bioreactor setup and operational parameters for SCP production

To produce microbial SCP, four 4-L bioreactors were operated as sequencing batch reactors (SBRs) on continuous 12-h cycles with intermittent aeration for 136 days. Reactors received wastewater from a soybean processing company in Singapore as described in Vethathirri et al*.*^18^. Water temperature was maintained at 30 °C, and the sludge was continuously mixed at 375 rpm. The feeding phase occurred during the initial 5–10 min of a cycle, followed by 180 min anoxic/anaerobic and 540 min aerobic phases, after which the biomass was left to settle for 60 min and 1.35 L of the supernatant was discarded. Thereafter, the reactor was filled with the same volume of soybean wastewater, starting a new cycle. The pH was measured between 6.0 and 8.5, and the dissolved oxygen (DO) concentration was controlled between 0.2 and 0.5 mg L^−1^ during the aerobic phase. Each of the SBRs employed in this study was equipped with a magnetic stir plate to ensure mixed liquor homogeneity, a pair of EasySense pH and DO probes with their corresponding transmitters (Mettler Toledo), dedicated air and feed pumps, a solenoid valve for supernatant discharge, and a surrounding water jacket connected to a re-circulating water heater. The different portions of the cycle were controlled by computer software specifically designed for these reactors (VentureMerger, Singapore).

### Chemical analyses of fish diets and tissue

The nutritional composition, i.e., proximate composition (moisture, crude protein, crude fat, crude fibre, ash), amino acids profile, fatty acids profile, calcium, phosphorus, selenium, zinc, iron, and vitamins A, D, and B12 content of the control and experimental diets, as well as fish tissue (one sample per group) were analyzed according to standard methods^19^ by BV-AQ laboratories (Singapore).

### Microbial characterization of microbial community-based SCP

Three samples of 0.5 g each were collected randomly from a 2-L bottle containing 222 g of dry mixed-culture SCP to be used in the aquaculture trial. These were subjected to DNA extraction and as previously described^20^. Amplicon preparation was done by PCR using the primer set 341f/785r targeted the V3-V4 variable regions of the 16S rRNA gene^21^, as described in^22^. Bacterial 16S rRNA amplicon sequencing was done in two steps as described in Santillan et al*.*^23^. The libraries were sequenced on an Illumina MiSeq platform (v.3) with 20% PhiX spike-in and at a read-length of 300 bp paired-end. Sequenced sample libraries were processed following the DADA2 bioinformatics pipeline^24^. DADA2 allows inference of exact amplicon sequence variants (ASVs) providing several benefits over traditional clustering methods^25^. Illumina sequencing adaptors and PCR primers were trimmed prior to quality filtering. Sequences were truncated after 280 and 255 nucleotides for forward and reverse reads, respectively, the length at which average quality dropped below a Phred score of 20. After truncation, reads with expected error rates higher than 3 and 5 for forward and reverse reads, respectively, were removed. After filtering, error rate learning, ASV inference and denoising, reads were merged with a minimum overlap of 20 bp. Chimeric sequences (0.03% on average) were identified and removed. For a total of 3 samples, on average 26,426 reads were kept per sample after processing, representing 52% of the average input reads. Taxonomy was assigned using the SILVA database (v.138)^26^.

### Growth performance and statistical analyses

The individual growth performance of the fish was evaluated by calculating the mean weight gain (mean final weight − mean initial weight), percent weight gain (mean weight gain × mean initial weight^−1^ × 100), specific growth rate (SGR = (ln_mean final weight_ − ln_mean initial weight_) × trial duration^−1^ × 100), feed conversion ratio (FCR = mean feed intake × mean weight gain^−1^), and survival rate. Univariate analysis of growth performance parameters were done via Welch’s t-test assuming unequal variances in R (v.3.6.3). All reported P values for statistical tests in this study were corrected for multiple comparisons using a false-discovery rate (FDR) of 5%^27^. Heat maps for bacterial relative abundances of the SCP were constructed using the DivComAnalyses package (v.0.9) in R^28^.

### Preprint

A previous version of this manuscript was published as a preprint.

## Results and discussion

Both the control fishmeal-based diet (FM) and the microbial community-derived SCP experimental diet (SCP50) met the protein (> 45%) and fat (> 12%) requirements for juvenile Asian sea bass (Table 1). They were also formulated to provide essential amino acids to fish; however, at 18% and 14% in the FM control diet and 50SCP experimental diet, respectively, lysine was below the required level (Table 2). The remaining essential amino acid requirements were met, in agreement with a recent study by Vethathirri et al*.*^18^ where microbial community-derived SCP biomass produced from soy bean FPWW in lab-scale reactors was reported to fully or partially meet the essential amino acids requirements for fish and shrimp.

**Figure 1:**
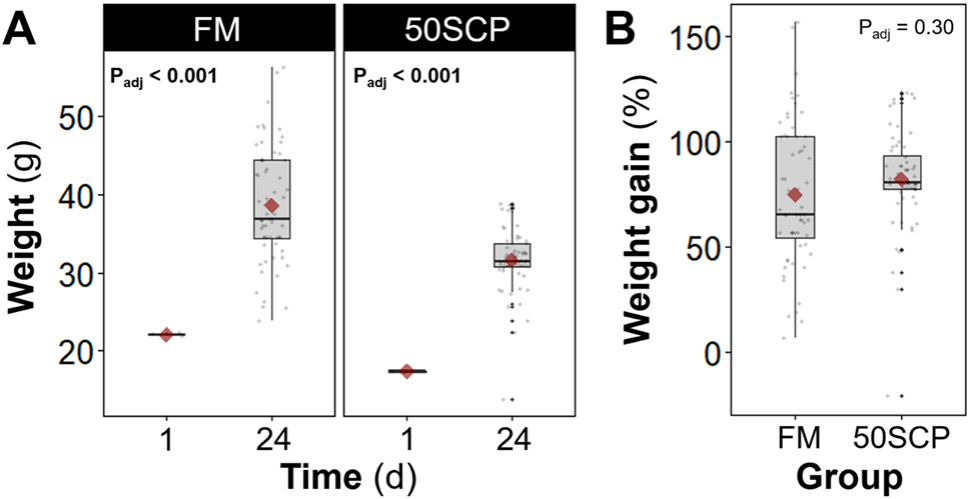
Weight gain of juvenile Asian sea bass (*Lates calcarifer*) fed with a fishmeal-based (FM) control diet or an experimental diet with 50% fishmeal replacement with microbial community-based SCP (50SCP) over 24 d. Panels represent (**A**) initial and final fish weight, and (**B**) percent weight gain per group. The box bounds the interquartile range (IQR) divided by the median, and Tukey-style whiskers extend to a maximum of 1.5 times the IQR beyond the box. Red diamonds display mean values. Grey dots represent values for each individual fish (n = 20) in each of three replicate tanks per group on d24. Welch’s t-test P values adjusted at 5% FDR shown within panels. Initial values (d1) are averages for each tank, calculated by simultaneously weighing all the fish in each tank and dividing by 20.

No significant differences in growth performance were observed between fish fed with the FM control and those that received the experimental microbial community-based 50SCP diet (Table 3). The initial weight of the fish in the group fed with the 50SCP experimental diet was significantly lower (Welch’s t-test P_adj_ < 0.001) than the control, despite the initial random assignment, but no significant difference was observed in the weight gain at the end of the feeding trial (Welch’s t-test P_adj_ = 0.30) (Table 3, Fig. 1). While a significant difference in initial fish weight between groups was possible due to the random assignment of individual fish and dietary treatments between tanks, this should not be a concern. The main variables analysed here were weight gain, feed conversion ratio (FCR), specific growth rate (SGR), and survival, all of which are independent of any differences in initial weights between the groups -which, again, were due to chance from random assignment-, but rather depend on weight gain. The feed intake of the group fed with the FM control diet was not significantly different from that of the group fed with the 50SCP mix experimental diet. Hence, no significant differences were observed for FCR and SGR. No mortality was observed during the feeding trial in both groups (Table 3). Furthermore, the group fed with the FM control diet presented a 4.4- and 10.3-fold greater variability in percent weight gain and FCR than the group fed with the 50SCP experimental diet, respectively, suggesting that an SCP replacement diet could also lead to more homogeneous growth of farmed Asian sea bass. Exploring the specific molecular and physiological mechanisms responsible for the observed homogeneous fish growth is a promising avenue for future research.

**Table 3:** Growth of juvenile Asian sea bass (*Lates calcarifer*) fed with a fishmeal (FM) control diet or an experimental diet with 50% fishmeal replacement with microbial community-based SCP (50SCP) over 24 days.

Parameter	FM	50SCP	P_adj_^†^
Initial weight (g)	22.1 ± 0.21	17.4 ± 0.14	< 0.001^A^
Final weight (g)	38.6 ± 5.14	31.6 ± 0.78	0.28
Weight gain (g)	16.5 ± 5.30	14.3 ± 0.88	0.69
Weight gain (%)	74.8 ± 24.7	82.1 ± 5.58	0.69
Feed intake (g)	19.8 ± 1.56	16.8 ± 0.73	0.15
Feed conversion ratio^§^	1.26 ± 0.31	1.18 ± 0.03	0.69
Specific growth rate (% day^−1^)^‡^	2.30 ± 0.57	2.50 ± 0.13	0.69
Survival (%)	100	100	–

Values are means ± s.d.m. (n = 3).

^†^Welch’s t-test P-values adjusted at 5% FDR.

^§^FCR = mean feed intake × mean weight gain^−1^.

^‡^SGR = (ln _mean final W_ – ln _mean initial W_)  × trial duration^−1^  × 100.

^A^Difference due to chance from random assignment. 120 juvenile sea bass of 19.7 ± 2.6 g were randomly distributed in rectangular glass aquaria (100-L water volume) at a stocking density of 20 fish per aquaria. Subsequently, three aquaria were randomly assigned to each dietary treatment (n = 3).

To our knowledge, ours is among the first fish feeding trials with microbial community-derived SCP in the feed at a relatively high fishmeal replacement level (50%) with microbial protein. Other studies have investigated the use of pure or enriched cultures of purple non-sulfur bacteria (PNSB) as aquafeed additives for the whiteleg shrimp (*Litopenaeus vannamei*) at protein replacement levels of 1.2–5.8%^29^ or 5–11%^17^, and for barramundi (*Lates calcarifer*) at 33–100% protein replacement levels^30^. These studies reported growth performances for different protein replacement levels that were comparable to that of commercial fishmeal^17,30^, as well as probiotic properties against *Vibrio* pathogens^17,29^. In our study, the nutritional composition of the fish tissue at the end of the trial was similar for the groups fed either FM control or 50SCP experimental diets in terms of crude protein, fat, most amino acids and fatty acids, as well as some vitamins and minerals (Table 4). Nevertheless, lower concentrations were observed in the fish tissue of the 50SCP experimental group for lysine (− 17.6%), vitamin A (− 68.8%), DHA (− 20.7%), EPA (− 16.4%), and total n-3 fatty acids (− 16.5%) than in the FM control group. Future studies should focus on refining SCP production processes (refer to Vethathirri et al*.*^14^ for design considerations), exploring dietary modifications, and assessing the long-term fish health implications. Evaluating the impact of SCP in feed on immune parameters and stress tolerance, alongside optimizing SCP production to increase essential amino acid content, can collectively enhance nutritional levels in aquafeeds.

**Table 4:** Nutritional composition (“freeze-dried” basis) of juvenile Asian sea bass (*Lates calcarifer*) fish tissue fed with fishmeal (FM) control diet or a fishmeal replacement diet containing 50% microbial community-based SCP (50SCP) over 24 d.

Composition	FM	50SCP	Method
Proximate composition (%)			
Moisture	7.5	8.4	L
Crude protein	56.0	55.0	E
Crude fat	23.0	24.1	I
Crude fibre	< 0.1	< 0.1	D
Amino acid composition (mg/100 g)			
Alanine	3560	3530	F
Arginine	3290	3230	F
Aspartic acid	5900	6480	F
Glutamic acid	6880	6670	F
Glycine	3890	3810	F
Histidine	1120	1140	F
Isoleucine	2030	1900	F
Leucine	3730	3510	F
Lysine	4090	3370	F
Methionine	1470	1410	F
Phenylalanine	2060	1990	F
Proline	2470	2470	F
Serine	2100	2000	F
Threonine	2180	2080	F
Tyrosine	1420	1350	F
Valine	2250	2180	F
Tryptophan	458	417	G
Total amino acids	49,570	48,260	F
Vitamin composition			
Vitamin A (mg kg^−1^)	0.32	0.10	A
Vitamin D3 (IU kg^−1^)	< 20	< 20	C
Vitamin B12 (µg kg^−1^)	0.19	0.16	B
Mineral composition			
Selenium (µg/100 g)	69.6	65.5	J
Calcium (mg/100 g)	3640	3650	K
Iron (mg/100 g)	4.34	4.37	K
Phosphorus (mg/100 g)	1040	1000	K
Zinc (mg/100 g)	5.24	5.23	K
Fatty acid composition (mg/100 g)			
Alpha linolenic C18:3n-3	312	308	H
Arachidic C20:0	75.3	74.8	H
Arachidonic C20:4n-6	199	167	H
Docosanoic C22:0	31.6	32.2	H
Butyric C4:0	< 4	< 4	H
Capric C10:0	< 4	< 4	H
Caproic C6:0	< 4	< 4	H
Caprylic C8:0	< 4	< 4	H
Dihomo-gamma-linolenic C20:3n-6	49.2	59.5	H
Docosahexaenoic C22:6n-3 (DHA)	1180	936	H
Eicosadienoic C20:2n-6	12.3	10.2	H
Eicosapentanaeoic C20:5n-3 (EPA)	439	367	H
Eicosatrienoic C20:3n-3	13.2	11.5	H
Eicosenoic C20:1 (total)	131	130	H
Eicosenoic(Gondoic) C20:1n-9	131	130	H
Erucic acid C22:1n-9	21.4	16.7	H
Gamma linolenic C18:3n-6 (GLA)	9.86	8.61	H
Heptadecenoic C17:1n-7	44.8	46.9	H
Lauric C12:0	14.2	14.7	H
Lignoceric C24:0	27.9	27.9	H
Linoleic C18:2n-6	3690	3790	H
Margaric C17:0	103	101	H
Monounsaturated fat (g/100 g)	8.17	8.92	H
Myristic C14:0	565	570	H
Myristoleic C14:1n-5	6.96	6.96	H
Nervonic C24:1n-9	39.3	35.1	H
Oleic C18:1n-9	7200	7930	H
Palmitic C16:0	5630	6080	H
Palmitoleic C16:1n-7	713	735	H
Pentadecanoic C15:0	80.7	81.8	H
Pentadecenoic C15:1n-5	14.7	17.6	H
Poly unsaturated fat (g/100 g)	5.97	5.76	H
Saturated fat (g/100 g)	7.78	8.30	H
Stearic C18:0	1210	1270	H
Total fatty acid (g/100 g)	22.0	23.0	H
Total Omega 3 (g/100 g)	1.94	1.62	H
Total Omega 6 (g/100 g)	3.95	4.03	H
Total Omega 9 (g/100 g)	7.39	8.12	H

^A^Methods for the Determination of Vitamins in Food—Recommended by COST 91 (1986).

^B^In-House No. F148 (Based on ELISA method).

^C^AsureQuality Method 5547, HPLC.

^D^In-house No. F1 (Based on Pearson's Chemical Analysis of Foods, 7th Ed., Pg. 16–18).

^E^AsureQuality Method 6503s, Kjeldahl Block Digestion.

^F^Analytical Biochemistry 178 (1989) (Modified).

^G^AsureQuality Method 5707, HPLC.

^H^AOAC 991.39.

^I^ sureQuality Method 4988, Gravimetric-SBR.

^J^AsureQuality Method 6311s, ICP-MS.

^K^AsureQuality Method 6324s, ICP-OES.

^L^AsureQuality Method 4208, Oven Dried.

**Figure 2:**
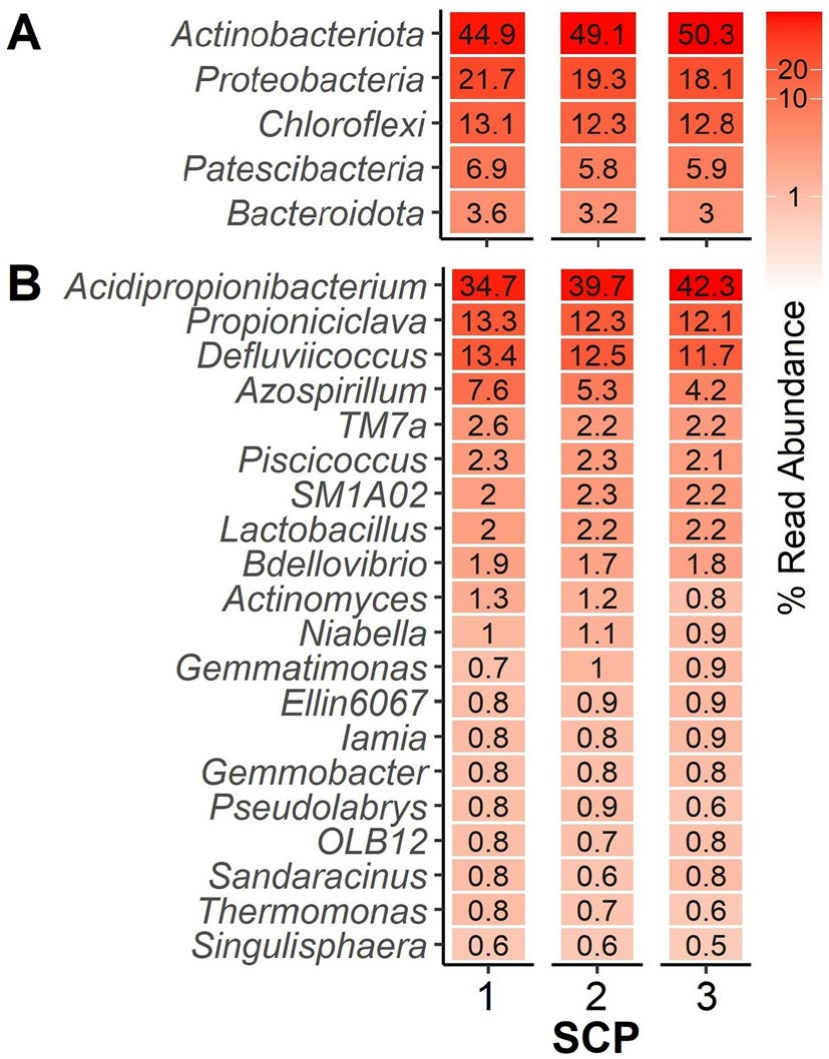
Characterization of the microbial community-based SCP produced from soybean processing wastewaters, assessed through 16S rRNA gene amplicon sequencing. Heat map displaying the relative abundances of the (**A**) top 5 phyla and (**B**) top 20 genera in three separate samples of the SCP mixture used in the study.

A microbial community-based approach to build SCP has several potential advantages over axenic culturing^14^, such as a higher protein content due to synergistic interactions between different SCP-producing microorganisms and the utilization of various carbon and nitrogen sources available in the substrate^18,31^, the accumulation of intracellular components^32^, and process stability in terms of resistance and resilience to disturbances^33–35^. The microbial composition of the microbial community-based SCP employed in the experimental diet was dominated by the genera *Acidipropionibacterium* and *Propioniciclava* (Fig. 2), which have potential as probiotics and producers of valuable metabolites. The stability and dynamics of *Acidipropionibacterium* and *Propioniciclava* in microbial cultures can be influenced by factors such as pH, temperature, and nutrient availability^36^. The presence of these genera and others from the family *Propionibacteriaceae* in the composition of microbial community-based SCP used for aquafeeds provides several potential advantages. Members of *Propionibacteriaceae* exhibit versatile metabolic capabilities and can thus ferment a wide range of substrates, contributing to the efficient utilization of diverse organic compounds during SCP production^37,38^. Furthermore, many *Propionibacteriaceae* are known for their ability to produce propionate through anaerobic fermentation^36^. Propionic acid is commonly used as a preservative in food and feed due to its antimicrobial properties. Other potentially probiotic properties^39^ include a positive influence on the gut microbiota of aquatic species, contributing to improved health, disease resistance, and overall well-being in aquaculture.

Taxa within the *Acidipropionibacterium* genus (previously named *Propionibacterium*) have a broad range of applications in the pharmaceutical and food industries due to their ability to produce vitamin B12, bacteriocins, and trehalose^40^. Indeed, there was 12 times more vitamin B12 in the experimental diet compared to the control (Table 2). Further, the species *Acidipropionibacterium acidipropionici*, which was detected in the 50SCP experimental diet, is used in the food industry as either a bio-preservative or probiotic due to its generally-recognized-as-safe (GRAS) status^41^. Some of these potential properties associated with a subset of the microbial community-based SCP could have contributed to the observed more homogeneous growth performance, in terms of FCR and weight gain, of the fish fed with the experimental diet compared to those fed with the control diet (Table 3). For example, Alloul et al*.*^17^ hypothesized that PNSB would be able to colonize the gastrointestinal tract and assist the digestion of white leg shrimp given that, similar to our study, the microbial protein used was freeze-dried, which allows for bacteria to be revived. However, how the microbial communities present in the experimental SCP diet interact with the indigenous gut microbiota of juvenile Asian sea bass, and to what extent these interactions contribute to the observed growth performance and probiotic potential, remains unknown. As the current knowledge of microbial community-based SCP as protein replacement in fish feed is limited, more research is needed to understand the underlying mechanisms behind our observations.

Based on the results of this preliminary trial, 50% of the fishmeal protein might be replaced with microbial community-based SCP meal without negatively affecting Asian seabass growth or survival in the short term. Additional research is required to address potential long-term effects on fish health, growth, and overall sustainability of aquaculture production, and whether these effects vary as a function of fishmeal protein replacement. Examining growth trajectories and weight gain over extended periods would help in understanding how fish respond to microbial community-based SCP as an alternative dietary component. Long-term studies could assess the efficacy of different dosages of the SCP on the immune parameters of the fish in addition to their growth performance. Moreover, other fish species and sources of FPWW could also be explored. It has been shown that microbial community-based SCP grown on wastewaters from potato and starch, brewery or dairy industries meets several of the essential amino acid requirements of trout and shrimp as reviewed by Vethathirri et al*.*^14^ and recently demonstrated for soybean processing wastewater^18^. However, FPWW of variable chemical composition may also lead to different mixed SCP communities^18^, thus more research is needed for a broader validation and implementation of this approach.

## Conclusions

This preliminary trial study showed that 50% of fishmeal protein might be replaced with microbial community-based SCP produced from soybean processing wastewater without affecting Asian seabass growth or survival, and that an SCP replacement diet may also lead to less variable fish growth than a traditional diet containing only fishmeal as protein source. Future studies should consider longer growth periods and higher fishmeal replacement levels, as well as additional aquaculture species and types of FPWW. Overall, we demonstrated that microbial community-based SCP has potential as an alternative value-added ingredient for aquaculture feed, which can help in the transition to a circular bioeconomy.

## Data Availability

DNA sequencing data are available at NCBI BioProjects PRJNA890376.
